# Monoolein Lipid Phases as Incorporation and Enrichment Materials for Membrane Protein Crystallization

**DOI:** 10.1371/journal.pone.0024488

**Published:** 2011-08-31

**Authors:** Ellen Wallace, David Dranow, Philip D. Laible, Jeff Christensen, Peter Nollert

**Affiliations:** 1 Emerald BioStructures, Bainbridge Island, Washington, United States of America; 2 Biosciences Division, Argonne National Laboratory, Argonne, Illinois, United States of America; University of Oulu, Germany

## Abstract

The crystallization of membrane proteins in amphiphile-rich materials such as lipidic cubic phases is an established methodology in many structural biology laboratories. The standard procedure employed with this methodology requires the generation of a highly viscous lipidic material by mixing lipid, for instance monoolein, with a solution of the detergent solubilized membrane protein. This preparation is often carried out with specialized mixing tools that allow handling of the highly viscous materials while minimizing dead volume to save precious membrane protein sample. The processes that occur during the initial mixing of the lipid with the membrane protein are not well understood. Here we show that the formation of the lipidic phases and the incorporation of the membrane protein into such materials can be separated experimentally. Specifically, we have investigated the effect of different initial monoolein-based lipid phase states on the crystallization behavior of the colored photosynthetic reaction center from *Rhodobacter sphaeroides*. We find that the detergent solubilized photosynthetic reaction center spontaneously inserts into and concentrates in the lipid matrix without any mixing, and that the initial lipid material phase state is irrelevant for productive crystallization. A substantial *in-situ* enrichment of the membrane protein to concentration levels that are otherwise unobtainable occurs in a thin layer on the surface of the lipidic material. These results have important practical applications and hence we suggest a simplified protocol for membrane protein crystallization within amphiphile rich materials, eliminating any specialized mixing tools to prepare crystallization experiments within lipidic cubic phases. Furthermore, by virtue of sampling a membrane protein concentration gradient within a single crystallization experiment, this crystallization technique is more robust and increases the efficiency of identifying productive crystallization parameters. Finally, we provide a model that explains the incorporation of the membrane protein from solution into the lipid phase via a portal lamellar phase.

## Introduction

**Figure 1:**
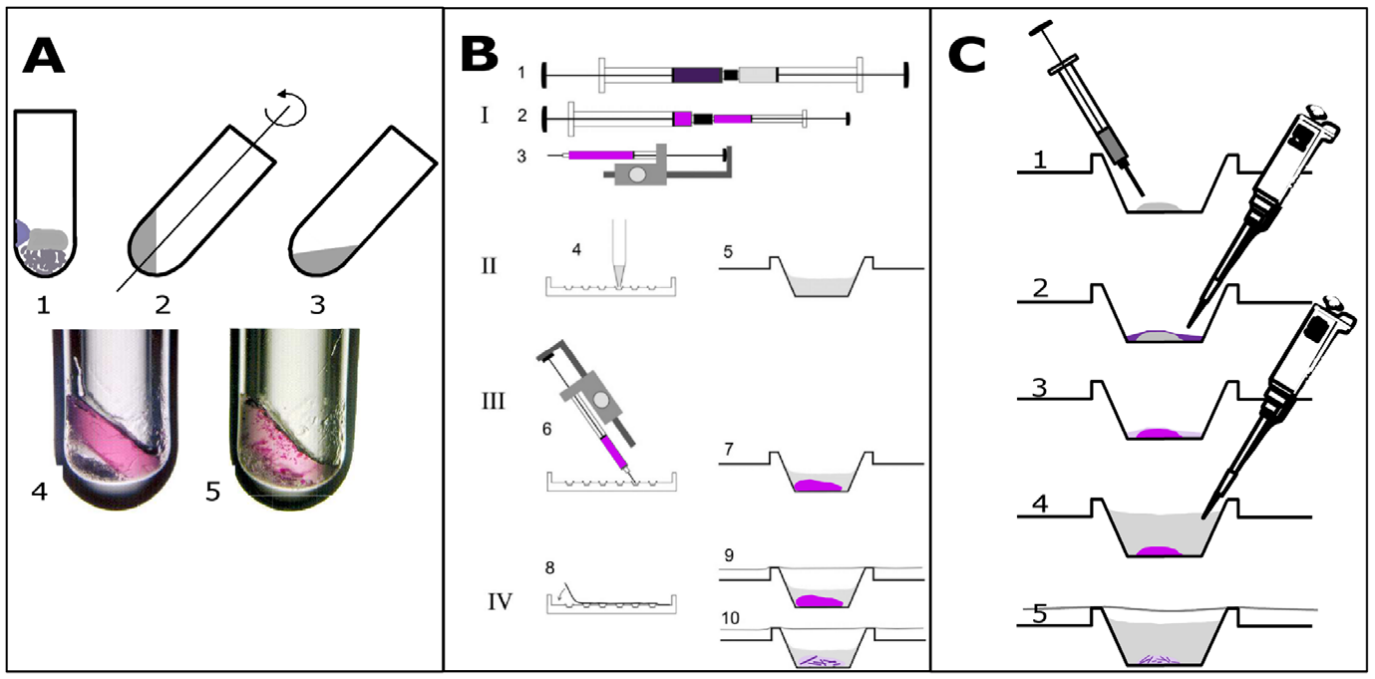
Brief diagrammatic history of the development of LCP-based crystallization techniques (∼15 years). A: Batch experiments carried out in micro test tubes [8]. Here, solid monoolein is combined with protein solution and precipitating reagents (1) and mixing is by 180-degree rotation of tube between centrifugation cycles (2,3). Each trial requires several microliters of protein and a minimum of 2 hours of preparation time (with typically a maximum of 24 simultaneous experiments); B: Syringe-based crystallization experiments where proteo-LCP is first prepared and then dispensed directly into precipitating reagents in crystallization trays, involving a four-step process: (I) Proteo-LCP is initially formed by coupling two syringes (I; one filled with 60% monoolein and the other with 40% protein solution) and by mixing of the two components with repetitive cycling of the entire combined volume from one barrel to the other. (II) Precipitant solutions fill the wells of a crystallization tray (4), a single well also shown (5). (III) Proteo-LCP is dispensed to each microwell with a semi-automatic ratchet dispenser (3, 6) after the material is transferred into a microsyringe (2). (IV) The experiments are sealed with clear transparent tape (8) and stored (9). The Proteo-LCP is stable in an excess of overlaying liquid (7). Crystals appear only within the lipid matrix (10). Proteo-LCP is dispensed into the precipitating reagents to avoid detrimental dehydration. A kit (Cubic LCP kit, Emerald BioSystems, Bainbridge Island, WA USA) and robotic versions of this dispensation technique [13] are available. Each experiment utilizes ∼200 nL of proteo-LCP – minimizing protein requirements and allowing for hundreds of precipitants to be screened simultaneously; C. PLI approaches, as adapted from [17], dispense fluid lipid materials into microwells using airtight syringes (1) prior to the addition of a solution of membrane protein by conventional pipetting (2). After a delay that allows the membrane proteins to integrate into the lipidic material (3), precipitating reagents are added (4) and the wells are sealed and stored (5). Here, the precipitating reagent dilutes the remaining unincorporated membrane protein solution. Crystals, again, only appear within the lipid matrix. PLI approaches also minimize protein requirements and are amenable to high-throughput approaches utilizing automated liquid handlers.

Lipidic cubic phases and related amphiphile-rich materials have served as matrices for growing a variety of membrane protein crystals [1], the latter of which were used in determining X-ray crystallographic structures of several high-impact target proteins such as G-protein coupled receptors [2], [3], [4], [5]. The procedures and tools employed to grow such membrane protein crystals have been refined over the past 15 years (Figure 1) and are used in many membrane protein crystallization laboratories [6], [7]. Initially, crystallizations were carried out as batch experiments in small test tubes with ca. 10 µL total setup volume [8], [9]. Soon after, a procedure employing positive displacement devices for the preparation of crystallization experiments in dedicated crystallization plates was introduced [10], later reproduced [11], [12], and refined with the goal to further reduce setup volumes and increase expediency [13]. Most of these technological developments aimed at improving the tools that manipulate small volumes of the highly viscous LCP (lipidic cubic phase) that is obtained when monoolein is mixed with a membrane protein solution [14]. Attempts have been made to avoid the requirement for dealing with the highly viscous LCP, such as devising protocols for crystallization within sponge phases [15], which are runny liquids that can be handled with standard laboratory pipettors, and were instrumental to produce crystals and a 1.86 Å crystallographic structure of the reaction center from *Blastochloris viridis* [15]. A drawback of this method is the requirement to add often undesired sponge phase-inducing reagents to crystallization experiments [16]. Recently, it has been reported that crystals of Photosynthetic Reaction Center from *Rhodobacter sphaeroides* and *Blastochloris viridis* can be obtained using a microfluidic device wherein the protein solution is mixed with an already established lipidic cubic phase material. This method was named PLI, post lipidic cubic phase formation incorporation [17].

Here we test different lipid phases that monoolein spontaneously forms with water and explore their utility in providing a matrix for membrane protein crystallization experiments. We aim to adapt the PLI preparation methodology to techniques that are compatible with standard laboratory liquid dispensation tools and practices (Figure 1). We also investigate the early stages of this new crystallization regime, namely the incorporation of the membrane protein RC (Photosynthetic Reaction Center from *Rhodobacter sphaeroides*) into a lipidic phase prior to crystallization.

## Results

### 1 RC Crystallization according to the PLI methodology

**Figure 2:**
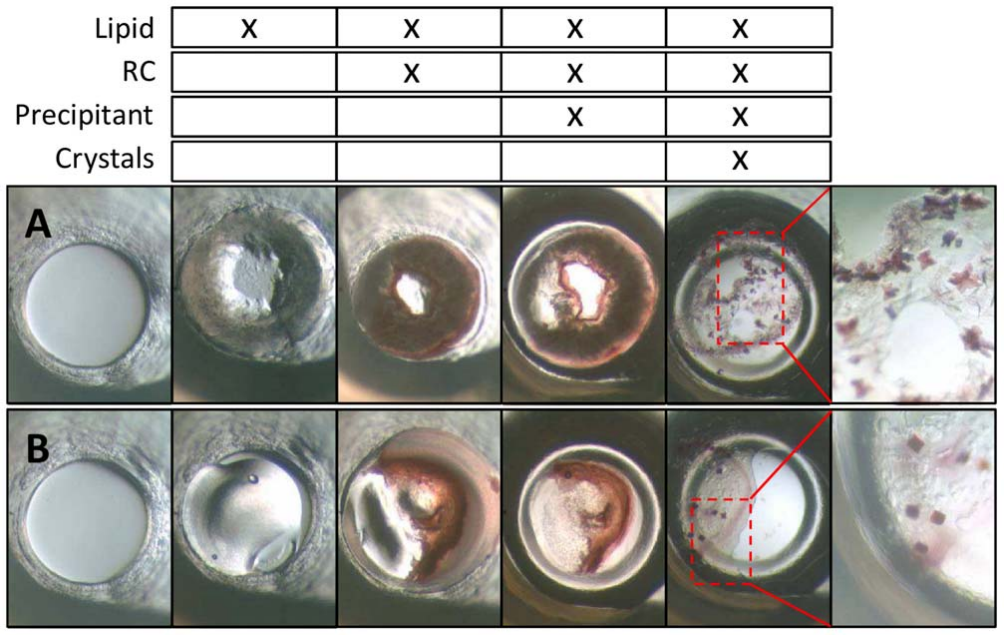
Images of the steps involved in conducting a PLI crystallization experiment with RCs using either neat, dry monoolein (image sequence A) or preformed LCP (40% water 60% monoolein; image sequence B). The process begins by adding 0.2 µl of the lipid or lipid mixture to the empty wells (1), of ca. 2 mm diameter, resulting in the second image in the series (2). Following sequential additions of RC solution (3; 0.4 µl) and precipitating solution (4, 2 µl in drop and 80 µl in reservoir), crystals were observed after 2 days (5). Magnified images of RC crystals are shown on the right. In these specific experiments, RCs were incubated with lipids for 4 hours prior to the addition of precipitating solution (1 M HEPES, pH 7.5, 1.15 M ammonium sulfate, Jeffamine M-600, 12% v/v). The top table tallies the components that are present at the time the images were taken.

**Figure 3:**
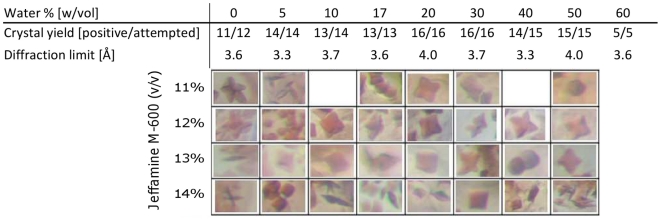
Yields of successful RC crystallization trials from independent PLI experiments where the initial monoolein hydration state and concentration of the precipitating agent, Jeffamine, were independently varied. The data represent results from highly replicated experiments and where RCs were allowed to incubate and integrate into lipid mixtures overnight prior to addition of precipitating solutions (which included 1 M HEPES/NaOH, pH 7.5 and 1.15 M ammonium sulfate in addition to Jeffamine, as indicated). Exemplary images of crystals observed 7 days after set up are shown for one particular replicate, each ca. 100×100 µm sections. Where images are absent, crystals were of poor quality or not observed for this particular trial. Crystal yield [positive/attempted] refers to the number of trials in which crystals were observed (positive) relative to the number of trials in which lipid, protein and crystallant all made contact (attempted). Diffraction limits were determined using an in-house X-ray source.

We have scaled up and adapted the PLI membrane protein crystallization methodology to be compatible with standard pipetting tools (Figure 1), as opposed to microfluidic devices [17], and applied it to the crystallization of RC. In fact, any monoolein lipid phase can be employed (Figure S1) to crystallize RC solubilized from LDAO (Lauryldimethylamine-oxide) which is first added to the lipid and then combined with precipitation reagents. In our experiments RC crystals grew and their X-ray diffraction limit was similar, regardless of the initial monoolein hydration level as long as monoolein was present (Figure 2, Figure 3, see also supplemental material, specifically Figure S2).

### 2 Optimized RC/monoolein pre-incubation time

**Figure 4:**
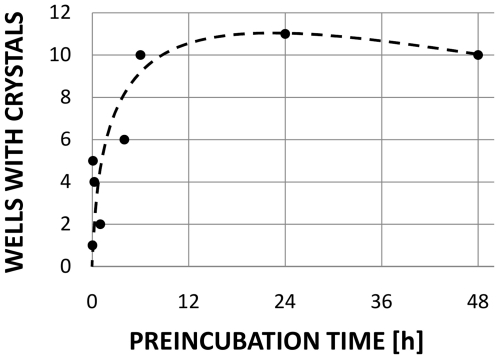
Effect of the length of RC/monoolein pre-incubation periods on the yield of productive crystallization experiments. Crystal yield is given as the number of successful experiments out of a total of 12 conducted for each pre-incubation period. Experiments utilized neat, dry lipid dispensed in molten form at 37°C. Crystallization success was judged 4 days after precipitant addition.

In order to devise a simplified PLI membrane protein crystallization protocol [17], we investigated the effect of the duration of the RC solution exposure to the lipid phase (Figure 4). We found that PLI setups yielded crystals for those experiments where the RC sample was incubated with monoolein for a time period of 2 hours to 2 days prior to addition of the precipitation reagent. The optimal incubation time was about half a day, conveniently carried out overnight. We noticed that the overall success of pre-incubation of RC with monoolein as compared to mechanical mixing of the protein solution with monoolein is substantially higher.

### 3 RC enrichment

**Figure 5:**
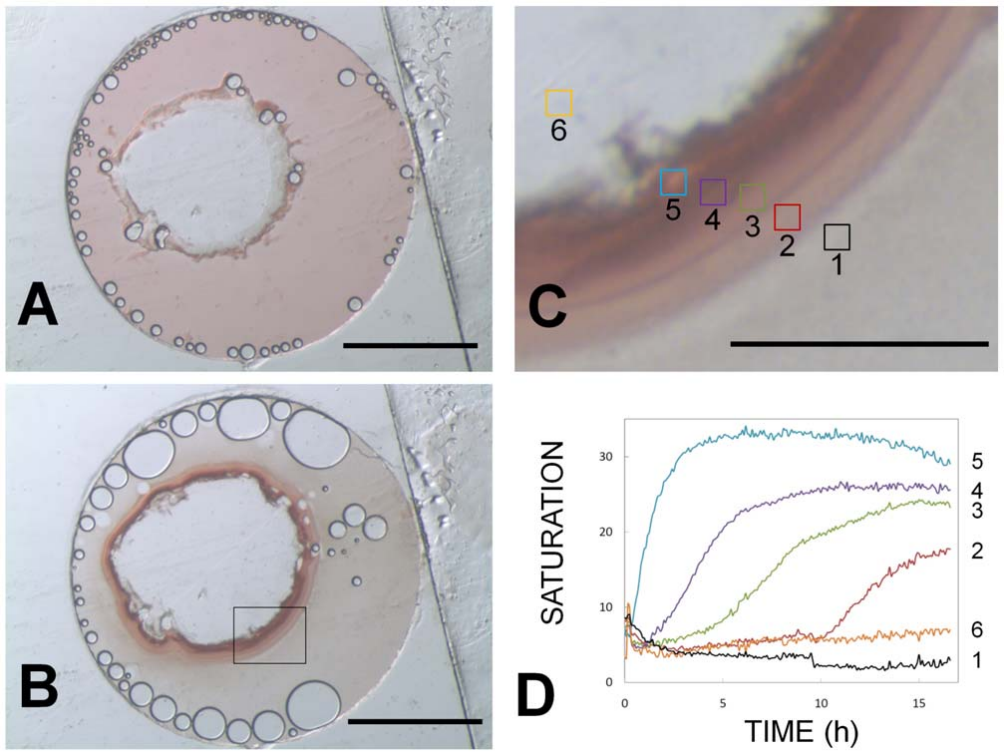
Tracking RC migration into the lipid matrix reveals the existence of concentration gradients. Here, the process of incorporation of RCs from solution into the bulk LCP is shown in a two-dimensional sandwich arrangement in the absence of precipitating solution. A: Initial image at ∼20 seconds post addition of 2.5 µl of RC solution (20 mg/ml) to a 0.4 µl bolus of LCP prepared with 44% water and 56% monoolein. Air bubbles from the RC solution preferentially adhere to the LCP (center) and transparent adhesive seal. B: Additional image after 16 hours of incubation. Here, RCs are depleted from the aqueous solution and enriched at the LCP/solution interface, and the central LCP area is devoid of RCs. RC concentrations may approach 146 mg/ml in the enrichment zone (7.3× enrichment factor) if the entire RC addition is localized to the area that the colored RC occupied at the interface. The observed 34% increase in area observed for the LCP matches that expected to occur as monoolein hydration increases from 44% to 58% (the latter is the maximum hydration of LCP at 16°C). Scale bar in A and B is 2 mm. C: Magnified image (scale bar  = 0.2 mm) of the six enriched zones that were monitored closely. RC concentrations were tracked in the bulk solution (Zone 1), the LCP/RC solution interface (Zone 2), and regions within the LCP at increasing distance from the LCP/RC solution interface (Zones 3, 4, 5, and 6). Color enhancement in Zone 4 is maximal at 16 hours and represents 3.3 times that of the initial color intensity of Zone 1 at the start of the experiments. Thus, there is an approximate 3-fold enrichment of RC concentration within the LCP in this zone. D: Quantitation of RC concentration using color saturation values of images, like those in A and B. Here, it is most evident that the concentration of RC in region 1 rapidly decreases and stabilizes at a minimum after ∼1 hour. The concentration of RC in the interior of the bulk LCP (region 6) increases only slightly throughout the experiment, indicating slow RC migration/equilibrium throughout the LCP. Zones 2 and 3 are initially part of the RC solution. These regions become enriched in RCs after 4 and 10 hours of incubation, respectively, as RCs migrate back to the aqueous liquid from the most rapidly- and highly-enriched Zones 4 and 5. Thus, after initially migrating directionally into the LCP and concentrating in Zone 5, the RCs subsequently migrate/diffuse freely in both directions (not only further to the interior of the LCP, zone 6, but also back towards the bulk aqueous solution). Zones 4 and 5 experience the largest increases in color saturation, with Zone 5 showing a distinct maximum at 5 hours, followed by a steady decline, possibly to the benefit of Zone 6.

In order to better examine the first step in the PLI process, incorporation of RC into LCP, we prepared thin sandwich setups similar to those described by Cherezov *et al.* [18] to enhance the optical inspection path through the setup, reduce aberrations and improve the interpretation of generated images. We took advantage of the chromophores within RC [19] to optically track the diffusion and concentration of RC in microscopy images by virtue of their red/purple color. The spontaneous enrichment of RC at the interface of dispensed LCP and detergent solubilized RC solution is evident from the darkening of the ca. 0.1 millimeter thick rim section around the LCP material (Figure 5) after exposure of the sandwiched LCP bolus to RC containing solution. Within minutes of initial exposure the RC color saturation and hence the RC concentration increases at this rim and reaches a peak after ca. 5 hours, corresponding to an approximate 3.3-fold enrichment within the lipid material at this location, as judged by the increase in color saturation. We wished to determine an independent estimation of this transient enrichment based on the difference in volume occupied by RC at the start and end of the experiment. Initially, the RC occupied an area of 14.47 mm^2^, calculated by the difference between the total area of the well (19.64 mm^2^) and the initial LCP bolus (5.17 mm^2^). The original area was compared to the final occupied area, which we calculated to be 1.98 mm^2^, based on the difference between the area defined by the outer ring of the bolus and the inner ring of the clear area of the bolus. This gives a fold increase of ∼7.3. For these calculations we chose to ignore the volume, as the sandwich plate creates a consistent height in all objects contained within. We suspect that these differences in these RC enrichment estimates are due to the inexact correlation of the image-based volume and color saturation with actual RC concentrations. Further, more quantitative studies are required to accurately assess the transient concentration of the membrane protein close to the solution exposed LCP.

The interface displays phenomena that are related to a number of processes, including the insertion of RC into the lipid phase, the expansion of the lipid phase, and possibly, additional phase transition and optical effects that are due to refractive index differences and light scattering from surfaces and at interfaces. Our interpretation as presented above assumes that the measured color saturation changes are dominated by local concentrations of RC. While the traces in Figure 5D contain information regarding the kinetics of partitioning and diffusion, additional experimentation, with apparati designed for increased control of parameters, is required in order to quantitatively assess coefficients and rate constants for these processes.

Nevertheless, the images clearly show that the RC enrichment at the rim section is of transient nature and involves the formation of an RC gradient towards the center of the LCP. At this rim, the initial increase in saturation is followed by a decline (Figure 5C, D). The final state of complete RC depletion in the solution and equilibrated RC throughout the bulk of the LCP is not reached during the timescale of a crystallization experiment, thus presenting an RC concentration gradient at the time of precipitation reagent addition. The ca. 0.1 mm thick rim section consists of several distinct, ca. 20–50 micrometer thick zones (Figure 5C), each exhibiting unique RC enrichment kinetics (Figure 5D). The comprehensive interpretation of the development of these zones is exacerbated by the dynamic nature of the rim, possibly caused by several simultaneous processes occurring during the course of the experiment. For instance, we observe an overall 34% hydration-triggered LCP expansion, the formation of distinct zones, and diffusion of RC. Importantly, the images of the rim section and their time dependent changes in color saturation clearly demonstrate that, within the timescale of a typical RC crystallization experiment, RC enriches to different levels within sections of the outer layers of the monoolein material (Figure 5C and D). During the course of incubation the reservoir of RC depletes in the aqueous solution, and the RC shows some equilibration within the bulk of the LCP by diffusion. The timescale of the enrichment is compatible with the diffusion of membrane proteins within LCP [20].

**Figure 6:**
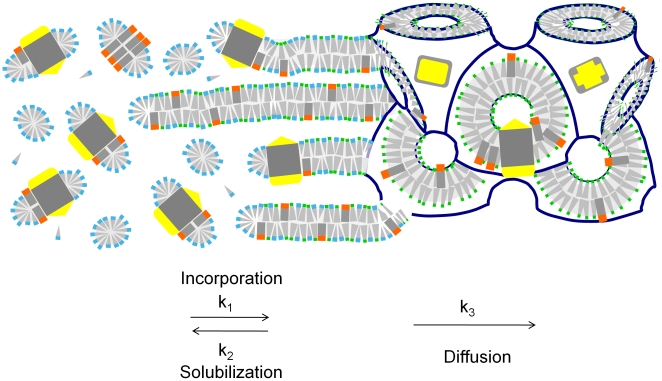
Illustration depicting how membrane proteins might incorporate into LCP in a PLI experiment. Solubilized membrane proteins (yellow and gray) are associated with native lipids (orange and gray) and are complexed into detergent micelles, the latter of which are in equilibrium with free detergent molecules (blue and gray). The relatively fast exchange of free detergent molecules with micellar structures allows for facile partitioning of detergent into the bilayer structure of the bulk LCP. The indicated k_i_ are time constants describing incorporation (k_1_), solubilization (k_2_) and clearance from the interface (k_3_). According to this model, productive incorporation from the micellar phase occurs if k_1_>k_2_ and interfacial concentration occurs only if diffusion is slow as compared to the incorporation step (k_1_>k_3_).Detergents have been shown to dramatically decrease the curvature of monoolein-based LCP [30], likely resulting in altered mesophase arragements of protruding bilayers consisting of monoolein (green and gray) and detergent molecules (blue and gray; in our case the detergent is LDAO) that serve as portals for membrane protein incorporation. These structures could promote the integration of membrane proteins into the curved, cubic, bulk material since they are extensions from that phase. Once assimilated, membrane proteins diffuse readily in LCP, with rate constants that are similar to those in planar bilayers, and are free to form nuclei and/or join growing crystals [20].

While the bulk monoolein cubic phase remained transparent and non-birefringent, the solution-exposed surface appeared shiny under microscopic inspection using crossed polarizers (not shown). We speculate that a thin section of the lipid material forms a portal lamellar phase at the solution exposed surface (Figure 6), allowing detergent and RC molecules to enter a bilayer structure that is connected to the curved lipid bilayer system within the LCP, a mechanism that is similar to the crystal growth hypothesis brought forward initially by [21] and later verified [22].

## Discussion

Our main result is that RC can be crystallized without mechanical mixing in monoolein-based matrices, regardless of the initial lipid phase state employed during pre-incubation. The presence of lipid bulk material is required for crystallization though, since RC crystals did grow only in lipid containing experiments. Hence, a specific interaction of the RC with the lipid phase is required for crystallization. We have shown that the interaction of RC with the LCP encompasses (i) a substantial transient RC enrichment at the LCP solution interface, and thus, (ii) the formation of a RC concentration gradient within the LCP during a timescale that is relevant for crystallization to occur. These phenomena are desired features in membrane protein crystallization experiments since the concentration effect increases the particle density, hence assuring supersaturation conditions within the crystallization experiment. Furthermore, the formation of a membrane protein concentration gradient within the matrix lipid constitutes an effective, continuous sampling of many different protein concentrations within a single crystallization experiment [23], [24]. The combination of these two features in the PLI-crystallization method [17] nicely explains the increased robustness and higher crystallization hit rate of ca. 25% as compared to standard LCP crystallization experiments, the latter of which require complicated pre-mixing of lipidic cubic phase materials [25].

Finally, the spontaneous insertion of the membrane protein from the detergent phase into the bilayer organization of the LCP is compatible with the current understanding of the mechanistic aspects (Figure 6) of crystallization of membrane proteins within lipidic cubic phases [21], [26]. We infer that the exposure of LCP to the RC solution initiates hydration of the LCP and fast partitioning of the detergent into the LCP. Detergents such as LDAO have been shown to form lamellar structures in ternary mixtures of monoolein, water and detergent [27]. While not observed directly, we assume that in our experiments LDAO initially enriches in an outer layer of the LCP as it partitions into the LCP, similar to RC as demonstrated in Figure 5. While the LDAO concentration in the bulk solution is not sufficient to convert the entire LCP into a lamellar phase, it is conceivable that its transient enrichment in the outer rim suffices to form membranous structures with low curvature. Such portal lamellar structures could form the entrance points for RC to fuse with and become part of the bulk LCP via diffusion (Figure 6).

The standard micro LCP crystallization method [10] (Figure 1B) is carried out by mixing the detergent solubilized protein solution with dry lipid, yielding an LCP with incorporated membrane protein within less than a minute. This substantially faster membrane protein incorporation into LCP using the syringe-based mixer method is presumably caused by the employed turbulent mixing regime, forming large interaction surfaces between lipid and solution, making the membrane protein incorporation process very efficient.

Taken together, the reported findings have several practical consequences: (i) Membrane protein crystallization with pre-dispensed lipidic phases are greatly simplified (Figure 1) because the handling of the lipid material and the membrane protein solution are separated processes that can be carried out independently of each other and with suitable dispensation tools. For instance, the dispensation of the highly viscous lipidic cubic phase with positive displacement syringes can be replaced by the dispensation of relatively low viscosity molten monoolein lipid. This enables the testing of very small quantities of precious membrane protein samples as these samples are not required to be mixed with coupled syringe devices (250 µl) bearing about a 5 µl dead volume, [10], [11], [12]. Hence, crystallization plates with pre-dispensed lipids can be prepared in advance and made commercially available (*i.e.* NeXtalCubicPhase uplate; Qiagen, Hilden, Germany). Indeed, crystals of Sensory Rhodopsin II (*H.Salinarum*) and that of an unidentified G-protein coupled receptor protein have been obtained using this approach (personal communication, Frank Schaefer Qiagen, http://www.qiagen.com/literature/render.aspx?id=104833). Within a relatively short period of time, productive crystal growth of four different membrane proteins has been reported with the PLI approach. Within a relatively short period of time, productive crystal growth of four different membrane proteins has been reported with the PLI approach. While we think this bodes well for the applicability of this protocol to membrane proteins in general, a careful comparative analysis of productive crystallizations is required to fully appreciate its utility. (ii) The initial lipid hydration extent, the period of incubation with the membrane protein solution and the crystallization setup geometry, specifically the size of the exposed lipid material surface area, add further crystallization optimization parameters to potentially improve the quality of membrane protein crystals. (iii) Similar to gel-based gradient crystallization methods [23], [24], the sampling of many different membrane protein concentrations in a membrane protein concentration gradient within a single setup enhances the efficiency and robustness of the crystallization experiment. Thus in primary screening experiments more parameters are sampled, enhancing the success rate to identify productive membrane protein crystallization conditions. (iv) Since the membrane proteins spontaneously concentrate at the lipid material surface, samples that would typically be considered unfit for crystallization experiments owing to their low protein content may be subjected to PLI crystallization trials. Indeed, we have demonstrated (data not shown) that RC crystals can be grown from diluted RC solutions (*i.e.* 2.5 mg/ml) using the PLI method. This is a significant advantage over traditional crystallization methods because the generation of membrane protein samples with high protein concentrations, typically exceeding 10 mg/ml, is often the main experimental barrier for membrane protein crystallization trials. The observed concentration factors of 3.3 to 7.3 relax this requirement substantially. Furthermore, this concentration effect may be used to enrich membrane proteins for purposes other than for crystallization, for instance for functional assays or storage. (v) Compared to mixing of LCP in coupled syringes where high shear stress is exerted on the lipid matrix and the membrane protein, the incubation of the membrane protein solution with portions of pre-dispensed lipid provide gentler reconstitution conditions, the latter of which may aid the application of labile membrane proteins to such crystallization trials. Hence it extends the crystallization optimization repertoire for those cases where mixing with monoolein destabilizes or renders the protein uncrystallizable [12], [28]. On the other hand, faster incorporation of the membrane protein, brought about by mechanical mixing, may be a gentler procedure for proteins that are less stable in the detergent phase than in the LCP at room temperature.

Aside from these practical aspects, the reconstitution of membrane proteins from a mixed membrane protein detergent complex and detergent micelle phase into a lipid bilayer system is of fundamental interest to membrane protein research. While the details of the membrane protein incorporation processes into the bilayer structure of an LCP remain poorly understood, we hypothesize that the partitioning of detergent into the LCP promotes the formation of lamellar structures that aid the insertion of detergent solubilized membranes into the bilayer structure of the LCP (Figure 6). We note that this new experimental format provides a simple system that allows dissecting the processes involved in membrane protein reconstitution. Unlike the homogenous reconstitutions in solution, this PLI system provides a heterogeneous experimental system with spacial fixation of the lipid bulk allowing for detailed investigation of processes that ensue during the incorporation of membrane proteins into membranes.

## Materials and Methods

### 1 Preparation of RC samples

Samples of *R. sphaeroides* RCs, solubilized and purified using the detergent LDAO were prepared as described in [25]. RC concentration was 20 mg/ml and solubilized in 10 mM Tris, pH 7.8, 0.05% (w/v) LDAO, 280 mM NaCl. Small aliquots of RC were shock frozen in liquid Nitrogen, stored at −80°C and thawed quickly prior to use [29].

### 2 Preparation and Characterization of Monoolein-based lipid phases

Monoolein phases were prepared by melting monoolein (Nu-Check, Elysian, Minnesota, USA) and mixing with water using a syringe-based apparatus as described [10]. In short, molten monoolein was filled into one RN-type 250 micro liter syringe (Hamilton, Reno, NV) and water was filled into a second syringe. The syringes were joined with a coupler (Emerald BioSystems, Bainbridge Island, WA) and a homogenous mixture was created by pumping the content of one syringe into the other, with more than 50 repeats. The final volume typically consisted of ca. 50–100 µl lipidic material; for example, to prepare a 30% v/w water/monoolein mixture one would combine 30 µl water with 40 mg monoolein. The optical properties of the obtained materials were assessed with and without crossed linear polarization filters (Figure 1). Only mixtures with 30% v/w, 40% v/w and 100% v/w water content in monoolein were transparent and non-birefringent, and all remaining phases were turbid or birefringent, as expected from isotropic lipid materials [30], [31], [32]. The rheological properties were crudely characterized by measuring the force required to pump the lipid material through the coupler from one syringe into another syringe. This was done by reading the weight measured when the syringe plunger of the assembly was placed onto a balance and the coupled syringe contraption was operated by pushing the plunger against the balance. Weight readings were taken when the resistance to push the plunger of the lipid filled syringe contraption was overcome. All lipid materials passed through the same syringe and coupler for all measurements. The highest resistance, found in the 30% v/w water/monoolein mixture, was set to 100%. The average standard deviation of such viscosity measurements was 8% (N = 30). Only lipid samples prepared with monoolein with 30% v/w and 40% v/w hydration displayed the hallmark properties of lipidic cubic phases: transparency, non-birefringence and high viscosity. The assignment of the obtained materials to their respective lipid phase type (Figure S1) is in perfect agreement with published monoolein phase diagrams [30], [31], [32]. While the monoolein phase diagram [33] shows stable LCP only for temperatures above 18°C, the materials we obtained showed all the hallmark properties of lipidic cubic phases. We speculate that such cubic phases form at slightly lower temperatures due to the presence of LDAO, sodium chloride and RC.

### 3 RC crystallization trials

RC crystallization experiments were carried out by adapting the crystallization recipes as previously described [1], [25], [34]. In short, ca. 0.2 µl of lipidic material were placed into a drop well of a crystallization plate at 16°C (Clover Jr. plate, Emerald BioSystems, Bainbridge Island, WA). To this, 0.4 µl RC sample were added to the lipid and incubated for various times. Following incubation, 80 µl precipitant solution were added to the reservoir, and from this 2 µl were transferred to the drop well, the plate sealed with transparent tape, and the plate incubated at 16°C. In the case of 0% protein/100% monoolein, the monoolein was melted (37°C) in order to aspirate it into a pre-warmed (37°C) ratchet dispenser, allowing repeated dispensation of supercooled monoolein in portions of 0.2 µl. All other phases were prepared via the syringe coupling apparatus (Figure 1B). Crystallization experiments were wrapped in foil to minimize exposure to light, and stored at 16°C. Experiments were inspected 48–72 hours after set up with a Leica MZ12.5 microscope. RC crystallization conditions consisted of an equally spaced one-dimensional, 4 condition screen with 1 M Hepes pH 7.5 and 1.15 M Ammonium Sulfate in the precipitate solution held constant, and Jeffamine M-600 concentration ranging from 11–14% v/v. The crystallization yields were computed from the hits from 5 to 16 replicates trials for each monoolein phase. While the RC preparation was capable of producing crystals in solution with crystallization reagents optimized for such growth [25], RC crystals did not form in the absence of any monoolein lipid (not shown) using the precipitation reagents employed.

### 4 X-ray diffraction of RC crystals

RC crystals were harvested directly from the wells and flash-cooled in liquid Nitrogen without further cryoprotection. RC crystals were subjected to maximum 30 second X-ray radiation using an in house Rigaku FR-E+ Superbright X-ray generator, Varimax HF optics, and a Rigaku Saturn 944+ detector. The highest resolution X-ray diffraction spots were assigned manually and were used to identify the resolution limit for each RC crystal tested.

### 5 Incorporation experiments using the sandwich format

A portion of LCP was prepared as described [10] by mixing monoolein with 44% (v/v) water at 16°C, yielding a transparent, non-birefringent and highly viscous material. 400 nl of LCP were dispensed into the center of a Laminex sandwich plate (Molecular Dimensions, Suffolk, UK). Around the LCP slug 2.5 µl of RC solution at 20 mg/ml were pipetted. A glass cover slip was attached to seal the well and to establish contact of the protein solution with the LCP. The well thickness was 100 micrometer. The setup was placed under a Leica MZ12.5 microscope equipped with an SPOT Insight 2MP Mosaic camera (Diagnostic Instruments, Inc., Sterling Heights, MI) and illuminated with a Volpi NCL 150 light source operated on the level 3 low setting. After fixing exposure parameters and white balance, images were recorded starting approx. ½ minute after assembly and then every 5 minutes for a total of ∼16 hours. Images were analyzed using ImageJ [35]. The scale in the images was approximated by using the diameter of the well (5 mm) as a reference length. RC concentrations were approximated by saturation levels that were computed by employing RGB values and the ImageJ function “Save XY coordinates” using the formula saturation  =  (max-min)/max·100. For each region of the image analyzed, a 144-pixel area was selected and the corresponding saturation values were averaged.

## Supporting Information

Figure S1 **Materials properties (transparency, birefringency, and viscosity) of the monoolein-based lipid solutions employed in RC crystallization experiments using the PLI approach.** Different lipid phases were created in syringe barrels by mixing solid monoolein with water. Water content labels (w/v fractions) are used to align images and tabulated data. A: Images of transilluminated syringe barrels with clear and/or turbid materials. B: Images of syringe barrels sandwiched between two crossed, linear polarizers (note that the background between the barrels is black, indicating complete light extinction). Lower Panel: Tabulated transparency ‘scores’ (N  =  no, not transparent; Y  =  yes, transparent), birefringence ‘scores’ (N  =  no, not birefringent; Y  =  yes, birefringent; S  =  some birefringence), and relative viscosity results. All lipid materials utilized display properties that conform to materials used in previous studies [ref] of monoolein phase behavior at room temperature.
(TIF)

Figure S2 **Representative X-ray diffraction results of RC crystals grown by the PLI method.** Shown are screenshots with X-ray diffraction images representing initial monoolein hydrations of 5% and 50%, depicting the best (A, B) and worst (B, C) diffraction. In order to show low and high resolution diffraction spots the diffraction images are shown in pairs A, B and C, D, each with low and high contrast setting, respectively. X-ray diffraction limits are listed in Fig. 3. Diffraction images were acquired with a CCD area detector (Saturn 944+) using a rotating copper anode X-ray source (Rigaku FR-E+). Rotation range was 0.5 deg, exposure time 60 sec.
(TIF)
