# Supplementary figures and images for: Monoolein Lipid Phases as Incorporation and Enrichment Materials for Membrane Protein Crystallization
Source: PLoS One. 2011 Aug 31;6(8):e24488. doi: 10.1371/journal.pone.0024488 (PMC3164205; doi:10.1371/journal.pone.0024488)

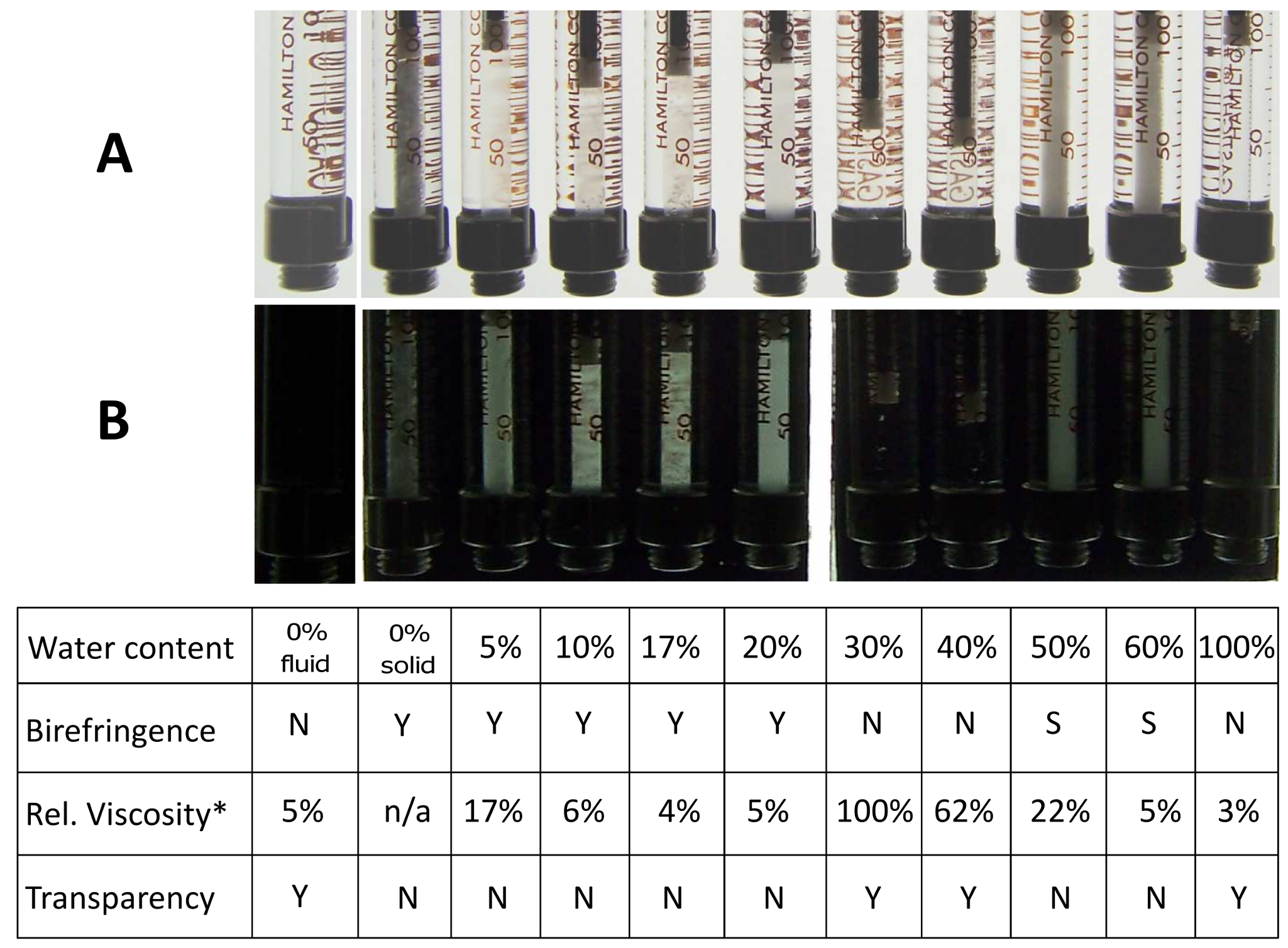

Supplement: Figure S1 — Materials properties (transparency, birefringency, and viscosity) of the monoolein-based lipid solutions employed in RC crystallization experiments using the PLI approach. Different lipid phases were created in syringe barrels by mixing solid monoolein with water. Water content labels (w/v fractions) are used to align images and tabulated data. A: Images of transilluminated syringe barrels with clear and/or turbid materials. B: Images of syringe barrels sandwiched between two crossed, linear polarizers (note that the background between the barrels is black, indicating complete light extinction). Lower Panel: Tabulated transparency ‘scores’ (N = no, not transparent; Y = yes, transparent), birefringence ‘scores’ (N = no, not birefringent; Y = yes, birefringent; S = some birefringence), and relative viscosity results. All lipid materials utilized display properties that conform to materials used in previous studies [ref] of monoolein phase behavior at room temperature. (TIF) [file pone.0024488.s001.tif]

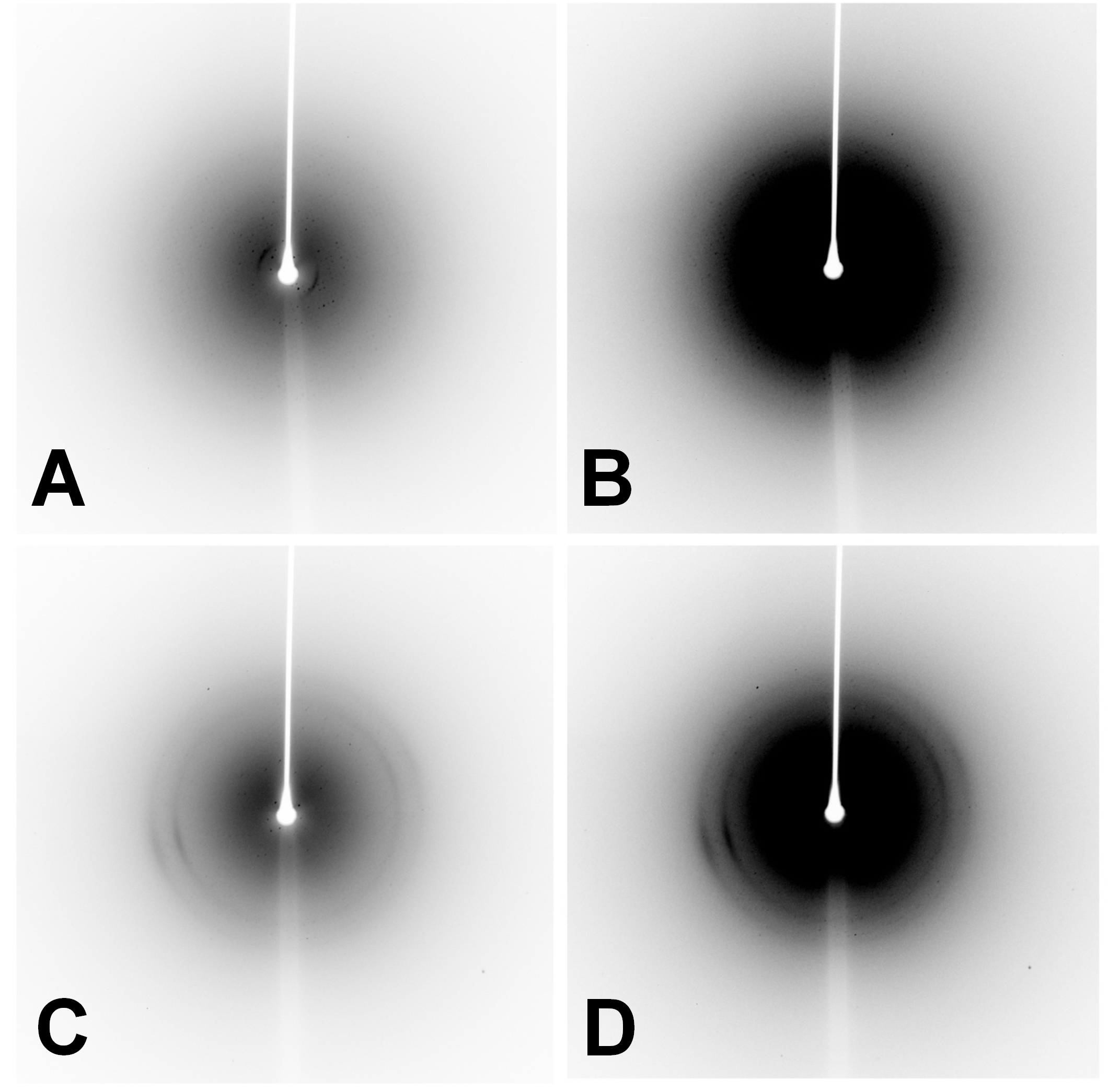

Supplement: Figure S2 — Representative X-ray diffraction results of RC crystals grown by the PLI method. Shown are screenshots with X-ray diffraction images representing initial monoolein hydrations of 5% and 50%, depicting the best (A, B) and worst (B, C) diffraction. In order to show low and high resolution diffraction spots the diffraction images are shown in pairs A, B and C, D, each with low and high contrast setting, respectively. X-ray diffraction limits are listed in Fig. 3. Diffraction images were acquired with a CCD area detector (Saturn 944+) using a rotating copper anode X-ray source (Rigaku FR-E+). Rotation range was 0.5 deg, exposure time 60 sec. (TIF) [file pone.0024488.s002.tif]
